# Extensive Metallosis in a Primary Knee Arthroplasty as a Result of Polyethylene Wear: Is It Avoidable?

**DOI:** 10.7759/cureus.57888

**Published:** 2024-04-09

**Authors:** Anas Bara, Abhimanyu Singh, Kuntal Patel, Deepak Herlekar

**Affiliations:** 1 Orthopaedics and Trauma, University of Central Lancashire, Preston, GBR; 2 Orthopaedics and Trauma, Royal Lancaster Infirmary, Lancaster, GBR

**Keywords:** wear, ceramic coating, polyethylene, total knee arthroplasty, metallosis

## Abstract

Metallosis is known to occur in metal-on-metal arthroplasty and has been of concern to orthopaedic surgeons worldwide. It is a rare, late complication of total knee arthroplasty (TKA), in which metal-on-metal contact leads to metal debris deposition in the surrounding tissue. Reasons for metal-on-metal contact could range from wear of the polyethylene insert to abnormal joint biomechanics. Many components can affect the development of metallosis, with polyethylene wear being the most common cause of metallosis. This paper discusses the case of an 85-year-old man who developed metallosis, attributed to polyethylene wear, 24 years after undergoing TKA. It also highlights the different components of knee prostheses, evaluates the efficacy of different types of polyethylene, and explores whether ceramic coating can improve TKA outcomes and reduce complications such as metallosis.

## Introduction

Osteoarthritis is the most prevalent joint condition, most commonly affecting the knee joint. It affects approximately 15% of individuals aged between 56 and 84 years [1]. In its advanced stages, osteoarthritis manifests through intense joint pain and deformities, with total knee arthroplasty (TKA) frequently emerging as the final effective intervention in managing this condition [1]. TKA became a highly popular procedure in the 1900s, producing outstanding outcomes in treating knee osteoarthritis. However, like every surgical procedure, it carries a risk of infection, implant loosening, osteolysis, and rarely, metallosis. Despite the rarity of metallosis, over the past decade, it has become a topic of interest [2]. Metallosis is characterised by abrasion between metal components within the knee joint, leading to the deposition of metal debris in surrounding tissues, and triggering inflammation. Metallosis leads to joint pain, instability, and swelling, which ultimately contribute to implant failure and associated complications [3]. Factors such as implant design, material composition, patient demographics, and surgical technique can all influence the risk of metallosis development [4]. This report discusses the case of an 85-year-old male who developed metallosis due to polyethylene (PE) liner wear following a TKA 26 years ago. PE is a thermoplastic material used in joint replacement surgeries owing to its durability, biocompatibility, and low-friction properties, which minimize complications such as wear debris-induced osteolysis and metallosis [5]. This case highlights the long-term implications of metal debris accumulation due to PE liner wear, even after decades of successful function.

## Case presentation

**Figure 1:**
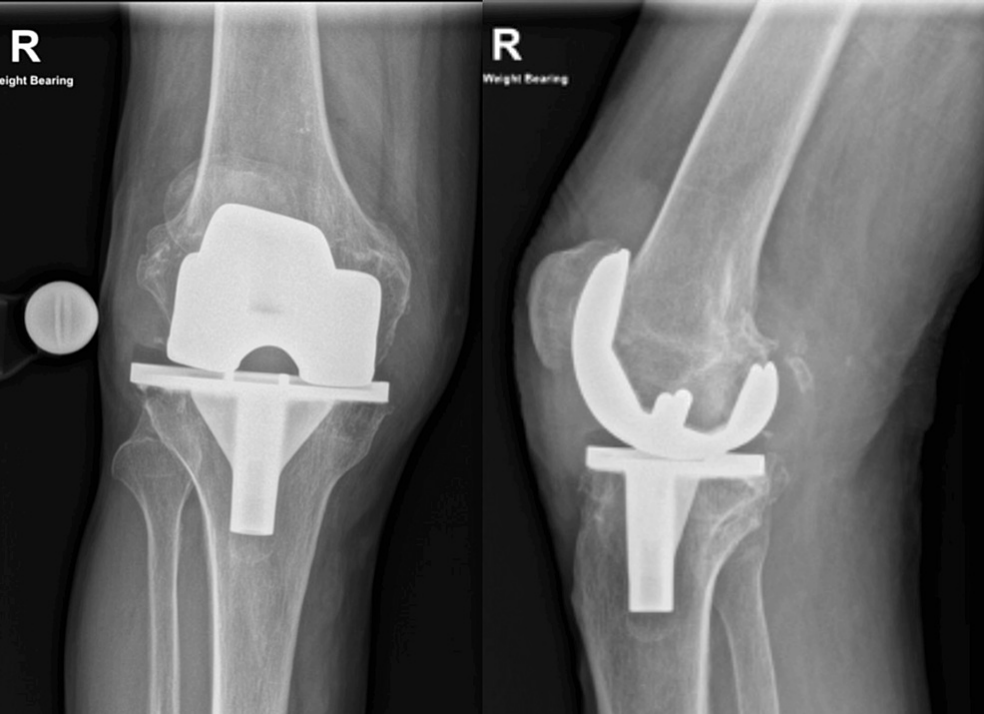
Anteroposterior and lateral X-rays of the right knee showing narrowing of the joint space.

An 85-year-old man, without any comorbidities, who underwent a TKA in 1996, presented to his General Practitioner in 2021 with knee pain and swelling. An X-ray showed PE wear and narrowing of the artificial joint space (Figure 1).

**Figure 2:**
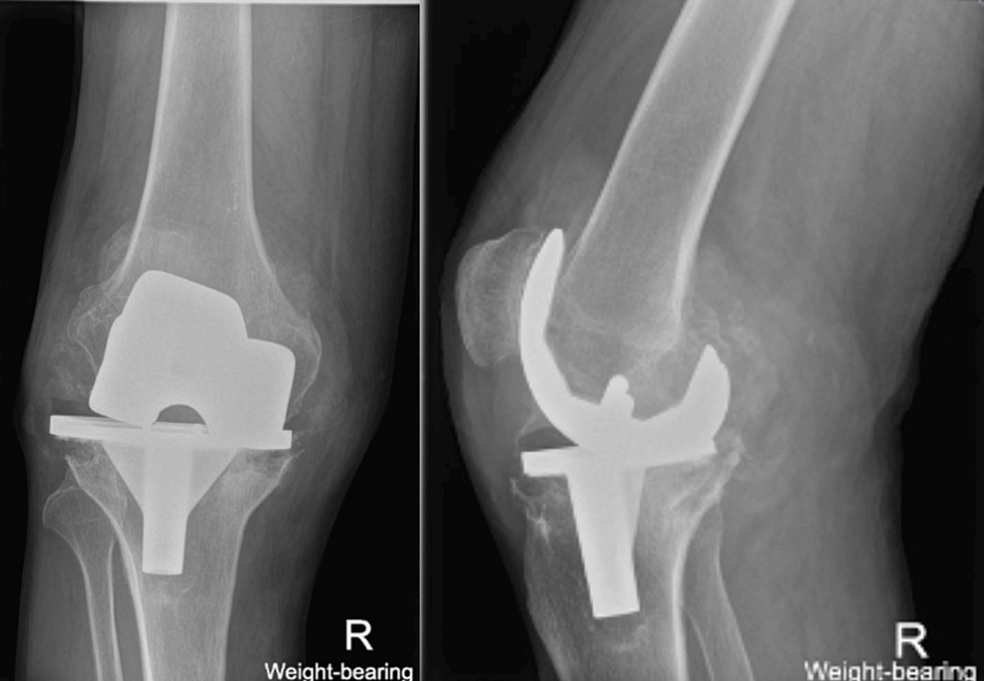
Anteroposterior and lateral X-rays of the right knee showing further wear of the polyethylene liner along with osteolysis.

However, due to the COVID-19 pandemic, follow-up and treatment were delayed. Two years later, the patient presented to us with increased knee pain, stiffness, swelling, decreased range of motion (ROM), crepitations, and having to rely on a stick for ambulation. Further imaging showed progressing PE wear, increased loss of joint space, and metal-on-metal articulation of the femoral and tibial components along with osteolysis (Figure 2).

**Figure 3:**
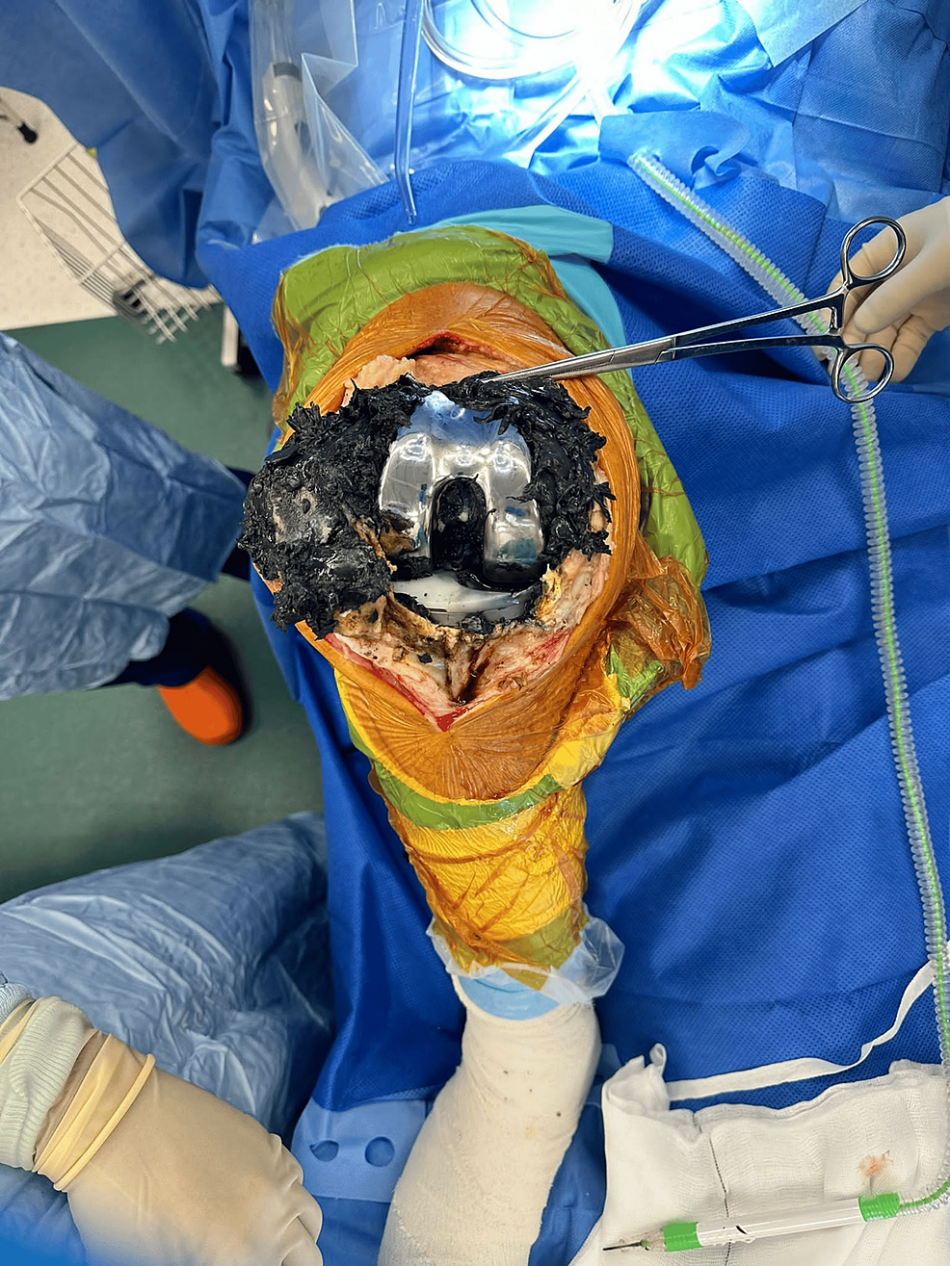
Metallosis within the surrounding tissue.

On clinical examination, the patient’s knee had a flexion deformity of about 15 degrees with further flexion to 90 degrees and no distal neurovascular deficit. The management plan involved conducting a revision surgery, during which all the implants would be removed from the right knee, followed by radical synovectomy of all inflammatory tissue with metallic debris deposits if present, and, finally, implanting a prosthesis with a level of constraint depending on the knee’s ligamentous integrity. During the surgery, extensive metallosis with dark black-stained synovial tissue was seen due to the deposition of metal debris in the surrounding tissue (Figure 3).

**Figure 4:**
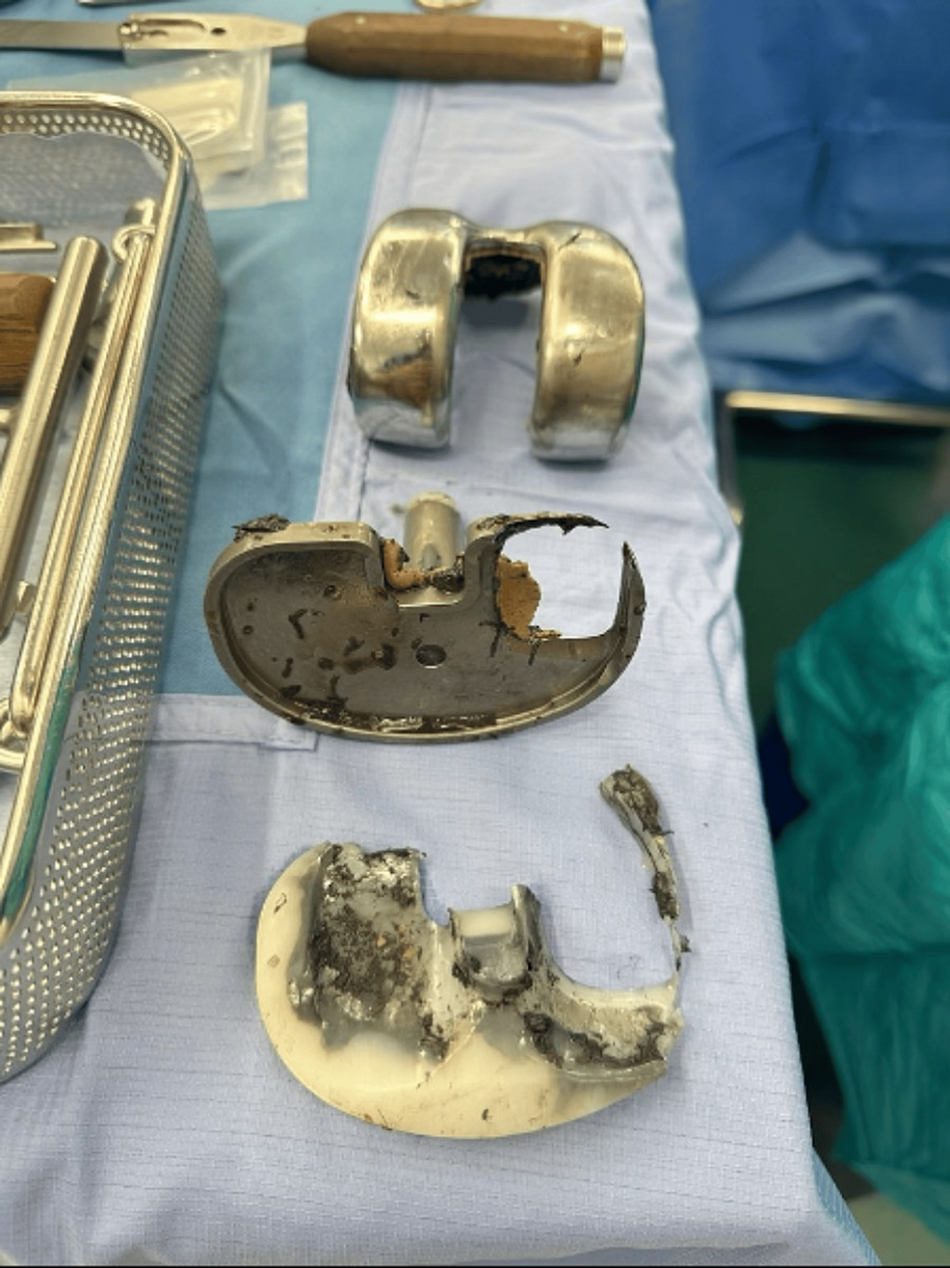
Knee prostheses of the right knee showing polyethylene wear with tibial tray defect.

**Figure 5:**
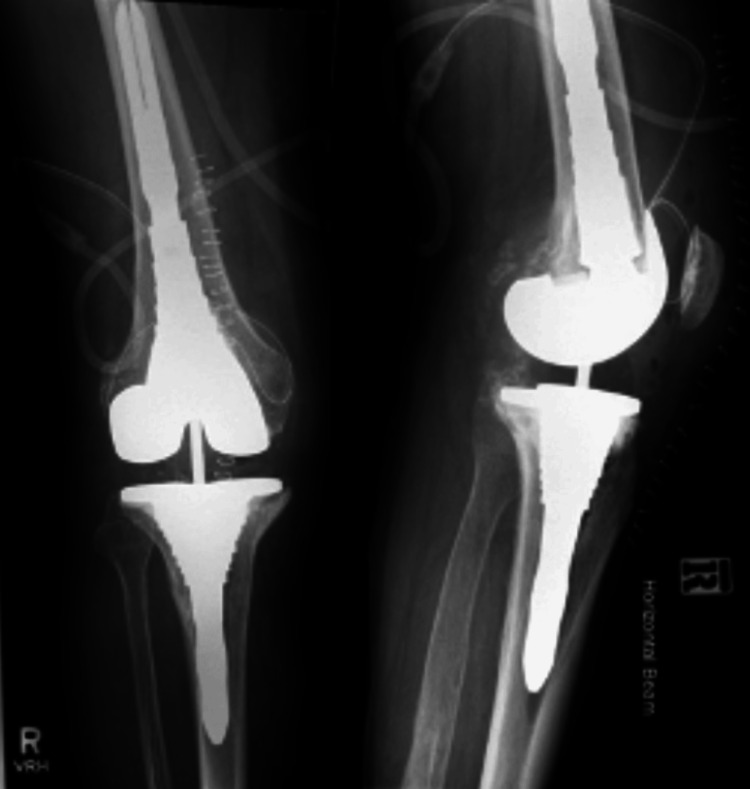
Postoperative anteroposterior and lateral X-rays.

**Figure 6:**
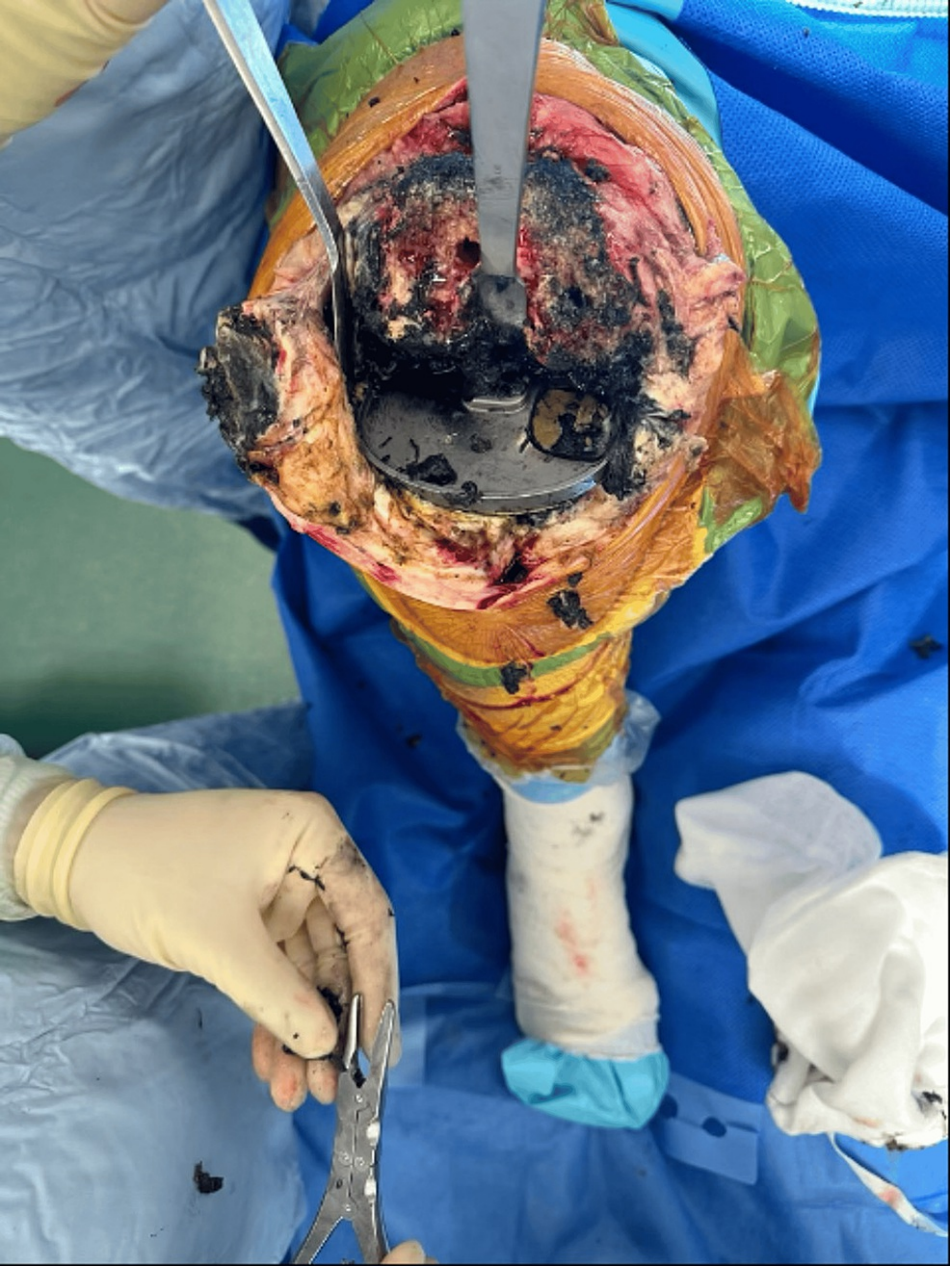
Tibial tray defect posteromedially.

After extensive debridement of stained tissue, the prosthetic components were removed (Figure 4), the knee was washed out, and a new semi-constrained modular revision knee prosthesis (Attune revision system, Depuy) was implanted (Figure 5) using sleeves and cones to achieve good fixation. The extracted prosthesis was a PFC (Press Fit Condylar, Depuy) Sigma fixed bearing, in which the femoral component was made of cobalt-chromium and the tibial tray was made of titanium with a matte articulating surface. The extracted insert was deficient posteromedially and resulted in a large defect in the corresponding area of the tibial tray (Figure 6). Because of bone loss and poor metaphysis, the sleeves and long stems were used on both the femoral and tibial sides. The patient had an uneventful postoperative recovery and was allowed to fully weight-bear. At his six-week follow-up, the scar had healed well and he was walking without any support with an ROM of 0 to 100 degrees.

## Discussion

In our case, extensive metallosis presented as a rare late complication of TKA. This complication occurred due to wear of the PE liner, resulting in metal-on-metal articulation and deposition of metal and PE debris within the surrounding tissue. Metallic debris infiltration in bone and soft tissue leads to osteolysis, tissue necrosis, and a pseudotumor. This eventually leads to pain, joint swelling, implant loosening, and instability. A comprehensive review revealed that PE wear/failure ranked as the most common cause of inducing metallosis due to its propensity for enabling abrasion between metal surfaces [6].

Another cause of PE failure contributing to metallosis is the design of knee prostheses [7]. The metal components of the knee prosthesis are stainless steel composed of cobalt-chromium-molybdenum (CoCrMo) alloy. The CoCrMo alloy has a large surface area exposed to the surrounding tissue, increasing the risk of metal debris and ion deposition in the surrounding tissue, resulting in an increased risk of metallosis [8]. Furthermore, with 15% of the population experiencing metal allergies, there is an increased risk of PE failure due to its potential to induce type 4 hypersensitivity reactions [7]. The introduction of ceramic nitride-based coatings such as titanium nitride (TiN) and titanium niobium nitride (TiNbN) offer a protective layer over the prosthetic components, solidifying the metal and minimising the release of metal debris into the surrounding tissue [8]. This is done through a specialized process known as physical vapour deposition, in which a 3-4 µm thick layer of TiN is applied to the outer surface [9].

A meta-analysis by Banci et al. [8] reviewed whether ceramic-coated knee prostheses offer superior outcomes compared to uncoated prostheses in primary TKA. The study concluded that ceramic-coated prostheses did not demonstrate superior outcomes compared to uncoated prostheses regarding the Knee Society Score (KSS), Oxford Knee Score (OKS), and complication rates. They also emphasized the necessity for further research to ascertain the cost-effectiveness of ceramic-coated and uncoated prostheses. Similarly, a study revealed that the 10-year survival rate of TiN-coated prostheses was very similar to that of uncoated TKA, indicating that the TiN coating offers no superiority [10].

Furthermore, in a five-year, double-blind, randomized controlled trial conducted by Van Hove et al., no clinical advantages were observed between TiN-coated and CoCrMo TKA in non-metal allergic patients [11]. Additionally, TiN components are suitable for patients with metal allergies, showing almost identical clinical and radiological results compared to those without metal allergies who received conventional implants [12]. In a rare case, a 64-year-old man developed metallosis four years after his TKA despite having ceramic-surfaced oxidized zirconium implants [7]. These studies highlight that although current evidence suggests limited clinical significance of coated versus non-coated knee prosthesis, in terms of survival rate, cost-effectiveness, and post-implant complications, further studies are necessary to explore the specific risk of metallosis between the two techniques and determine if ceramic coatings can effectively lower the risk of developing metallosis.

PE has been used in surgeries since the early 1900s reflecting ongoing efforts to improve the longevity, stability, and biocompatibility of these implants [5]. Initially, conventional PE (CPE) represented the first generation of PE implants. However, subsequent research revealed that wear rates were higher in young patients undergoing knee arthroplasty, attributed to their increased functional requirements. Consequently, this observation has encouraged the further development of different types of PE to reduce the risk of PE wear and osteolysis; these include vitamin E-infused PE (VEPE) and highly crosslinked PE (HXLPE). A 10-year follow-up control study conducted by Giustra et al. (2023) compared HXLPE and CPE in terms of clinical and radiological outcomes. It found that HXLPE has no additional benefits over CPE in preventing osteolysis, prosthesis loosening, infections, mechanical failure, or reduction in revision rates, all factors that can contribute to metallosis [13]. Moreover, HXPLE showed slightly higher ROM, KSS, and KSS functional scores but no significant differences. A study in 2009 compared 89 TKAs using HXPLE and 113 TKAs using CPE and concluded that there was no clinical difference in terms of the ROM, early implant failure, or any radiological outcomes between the two [14]. Hence, both Giustra et al. (2023) and Mindo et al. (2009) stated that the use of HXPLE over CPE in TKA remains controversial [13,14]. Furthermore, Takemura et al. (2019) performed a randomized controlled study comparing 100 TKA with CPE and another 100 TKA with VEPE. Similarly, their study concluded that after a two-year follow-up, there was no significant difference in ROM, KSS, and clinical and radiological results between the two types of PE [15].

Nevertheless, PE wear is closely linked to implant longevity, with wear debris contributing to aseptic loosening and failure. Therefore, minimizing wear is crucial. HXLPE, compared to CPE, may be more resistant to bacterial adhesion and the formation of biofilms [16]. Similarly, HXLPE shows a 26% lower revision risk for infection compared to CPE [17]. Interestingly, a study stated that HXLPE infused with vitamin E further decreased bacterial adhesion [15].

Ultrahigh-molecular-weight PE (UHMWPE) has not been widely used in TKA. Vitamin E infusion has been attributed to reduce oxidation and improve mechanical properties. UHMWPE was compared with vitamin E-stabilised UHMWPE in an ex vivo knee simulation test where each mechanical knee was simulated over 5 million times. The study concluded that vitamin E-stabilised UHMWPE wear was 73% to 86% lower than UHMWPE [18]. However, as this was a simulation, the practical limitations must be discussed, and a clinical test must be conducted to assess clinical outcomes.

## Conclusions

This case study highlights the presentation, management, and factors surrounding a rare yet significant occurrence of metallosis as a late complication of TKA, primarily attributed to PE failure. The evolution of bearing surfaces reflects ongoing efforts to enhance implant longevity and biocompatibility. However, no superior outcomes were found among VEPE and HXLPE, other than the reduced risk of infection. Moreover, the use of ceramic coatings in TKA is still controversial due to the limited evidence demonstrating significant clinical benefits compared to non-coated prostheses. Further research is needed to determine whether the specific risk of metallosis differs between coated and uncoated alloy implants. Finally, routine follow-up, especially when a decade has passed since the arthroplasty, can avoid such complications.
